# Serum Progesterone Profile Across the Mid and Late Luteal Phase in Artificial Cycles Is Associated With Pregnancy Outcome

**DOI:** 10.3389/fendo.2021.665717

**Published:** 2021-06-10

**Authors:** Elena Labarta, Cristina Rodríguez-Varela, Giulia Mariani, Ernesto Bosch

**Affiliations:** ^1^Reproductive Medicine Department, IVIRMA Valencia, Valencia, Spain; ^2^Research Department, IVI Foundation - IIS La Fe, Valencia, Spain; ^3^Reproductive Medicine Department, IVIRMA Roma, Roma, Italy

**Keywords:** progesterone, luteal phase support, ongoing pregnancy, endocrine profile, artificial cycle

## Abstract

**Introduction:**

Recent studies have shown that low serum progesterone levels on the day of embryo transfer (ET) are associated with poorer pregnancy outcome in hormonal replacement therapy cycles. It is of interest to know if serum progesterone levels during late luteal phase (following days after ET) are also related with the chances of ongoing pregnancy.

**Objective:**

To evaluate the luteal phase endocrine profile through measurements of serum progesterone and estradiol on days ET+4, ET+7 and ET+11, to test their predictive value in relation to pregnancy outcome.

**Setting:**

Private infertility center, Valencia, Spain.

**Materials and Methods:**

Prospective cohort study performed between June 2017 and August 2018. Eligible patients were aged between 18-42 years, with a normal uterus, and being transferred 1-2 good quality blastocysts in a frozen ET cycle after an artificial endometrial preparation with estradiol valerate and vaginal micronized progesterone (400 mg/12 hours).

**Results:**

A total of 127 patients were included. Mean age = 38.0 ± 3.9 years; BMI = 23.6 ± 3.6 kg/m2; endometrial thickness = 9.1 ± 1.6mm. Overall ongoing pregnancy rate = 47.2% (95%CI:38.3-56.3). Significantly higher levels of serum progesterone were observed on ET+4 (13.6 ± 6.0 *vs.* 11.1 ± 4.6ng/ml, p = 0.03) and ET+11 (15.7 ± 1.2 *vs.* 10.3 ± 0.6ng/ml, respectively; p = 0.000) in ongoing pregnancies *versus* negative β-hCG (β-human chorionic gonadotrophin) cases. On ET+7, ongoing pregnancies also had higher serum progesterone levels (14.2 ± 0.9 *vs.* 11.7 ± 0.8ng/ml, but did not reach statistical significance (p = 0.07). Serum estradiol levels were not related with pregnancy outcome at any moment of the luteal phase (p > 0.05). On days ET+4, +7 and +11, the ROC analysis showed that serum progesterone levels were predictive of ongoing pregnancy, and Pearson’s coefficient showed a significant association (p<0.05) of serum β-hCG levels with serum progesterone.

**Conclusions:**

In hormonal replacement therapy cycles, serum progesterone levels across luteal phase days are associated with pregnancy outcome. Ongoing pregnancies were associated with a higher exposure to progesterone in comparison with pregnancy losses or negative β-hCG. Therefore, serum progesterone might be playing an important role not only during implantation, but also in pregnancy maintenance. It remains unknown if the variability in serum progesterone levels among patients, after receiving the exact same progesterone dose for luteal phase support, is the cause or just a consequence of pregnancy results.

## Introduction

The interest on the impact of serum P levels during window of implantation has emerged in the last years. There seems to be a relationship between low serum P levels and poorer outcome, in terms of ongoing pregnancy or live birth rate (1–6). Most of the studies have been conducted in hormonal replacement therapy cycles, in which there is no endogenous progesterone -due to the lack of ovulation, and luteal phase depends exclusively on the amount of exogenous P given to the patients.

When using the vaginal route for progesterone administration, ongoing and live birth rates are significantly decreased when serum P is low on the day of embryo transfer, or even one day before/after (1–6). Prospective studies found that the critical threshold of serum P on the day of ET is about 9 ng/ml (5, 6). This negative impact remains present after adjusting for the most important variables, such as origin of oocytes (own or donated) or embryo quality.

Endogenous production of P during pregnancy might occur around week 6, when the uteroplacental shift occurs (7). Thus, levels of serum P in the mid-late luteal phase transition in artificial cycles could depend exclusively on the exogenous drug that is given to the patient.

The well described correlation between pregnancy outcome and levels of serum P on the days surrounding the ET might suggest a direct impact on embryo implantation. The remaining question is if this effect is also maintained during the late luteal phase, once the window of implantation is closed. If so, serum P would not only play an essential role on implantation potential, but also on pregnancy maintenance.

We wanted to elucidate if serum P levels on the days 4^th^, 7^th^ and 11^th^ following embryo transfer, were also related with the pregnancy outcome. Information about levels of estradiol according to the pregnancy outcome is also added.

## Materials and Methods

This is a parallel analysis of a prospective proof-of-concept single-center cohort study (termed hyperPOC study) performed between June 19th, 2017 and August 10th, 2018 in IVI Valencia, Spain. The study protocol received Ethics Committee and Institutional Review Board approval. The objective of the hyperPOC study was to determine if serum β-hCG measured on Day 4,7 and 11 after IVF-ET of Day 5 blastocysts was superior to the Elecsys^®^ HCG+β assay for predicting pregnancy in IVF-ET cycles. Additionally, P and E2 were also analyzed in serum on the same days after ET.

All patients provided their written informed consent. The clinical trial registration number is NCT03184519.

In this analysis we included those patients undergoing a hormonal replacement therapy cycle for embryo transfer (n=127). Eligible patients were aged between 18-42 years, with a normal uterus in 2D ultrasound, and being transferred 1-2 good quality blastocysts in a frozen embryo transfer after an artificial endometrial preparation with estradiol valerate and vaginal micronized progesterone (400 mg/12 hours) as described elsewhere (6).

We sought to evaluate potential differences in the hormonal profile (progesterone and estradiol) throughout the late luteal phase (day 4, 7 and 11 after ET) according to the pregnancy outcome. We analyzed also the predictive value of serum P during the late luteal phase; and correlated with serum β-hCG levels.

Pregnancy outcome was determined by a positive β-hCG test (serum levels of β-hCG>10 mIU/ml, 11 days after ET); clinical pregnancy was defined as the presence of at least one gestational sac on ultrasound; miscarriage rate was defined as any pregnancy loss before week 12, including biochemical miscarriage with a positive β-hCG test without evidence of a gestational sac and clinical miscarriage after confirmation of an intrauterine gestational sac; and ongoing pregnancy was defined as the presence of at least one viable fetus beyond week 12.

### Endocrine Assays

Serum progesterone and estradiol levels were measured three times during the mid and late luteal phase (on the 4^th^, 7^th^ and 11^th^ day after ET). In addition, progesterone levels were also measured on the ET day, following our routine clinical practice. Blood was drawn 6 hours after last insertion of vaginal P. Hormonal measurements were blinded, and the results were not available until the end of the study.

Blood samples were analyzed by an electrochemiluminescence immunoassay (CobasVR e411 analyzer, Roche diagnostics GmbH, Germany). Intra- and inter-assay coefficients of variation for the P determinations were 1.2–11.8% and 3.6–23.1%, respectively, for P-values between 0.22 and 51.6 ng/ml. Sensitivity was 0.03 ng/ml. The intra- and inter-assay coefficients of variation for estradiol determinations were 2.4–9.5% and 2.5–11.9%, with a measurement range of 25.4–2932 pg/ml. Sensitivity was 5 pg/ml.

Elecsys HCG+β was analyzed at Roche Diagnostics using a CobasVR e 411 analyzer, with a functional assay sensitivity of <0.6 mIU/ml.

### Statistical Analyses

Statistical report has been performed under R version 4.0.2 (2020-06-22) package software. An exploratory analysis has been performed to describe clinical and anthropometric data, as well as serum hormonal levels throughout late luteal phase. For each hormonal parameter, an ANOVA F-test has been performed to evaluate the homogeneity among pregnancy outcomes per day analyzed. A post-hoc analysis was performed to evaluate the correlation between paired variables within a significant previous multiple comparison by the ANOVA F-test. A linear regression model has been performed to evaluate the co-dependence between luteal phase days, for each one of the hormones analyzed. Correlation within each luteal phase day between ongoing pregnancy rate (OPR) and P has been analyzed under logistic regression models and validated through receiver operator characteristics (ROC) analysis. The optimal threshold was defined according to sensitivity and specificity to predict OPR.

## Results

A total of 127 patients were included. All of them were transferred a good quality blastocyst under an artificial cycle. Mean age of patients was 38.0 ± 3.9 years, with a BMI of 23.6 ± 3.6 kg/m2 and an endometrial thickness in the proliferative phase of 9.1 ± 1.6 mm.

Origin of oocytes were own in 60.6% and donated in 39.4% of the cycles. The mean serum levels of estradiol and progesterone in the follicular phase was 218.4 ± 120.4 pg/ml and 0.09 ± 0.09 ng/ml, respectively.

The overall positive β-hCG was 69.3% (95% CI = 60.5-77.2), with an OPR of 47.2% (38.3-56.3). Biochemical miscarriages occurred in 17 of the 88 positive β-hCG cases (19.3%; 95% CI = 11.7-29.1), while clinical miscarriages occurred in 11 of the 71 clinical pregnancies (15.5%; 95% CI = 8.0-26.0).

No differences were observed in the basal characteristics (BMI, EMT, estradiol or progesterone on the proliferative phase) between own and donated oocytes, except for the age, which was significantly higher with donated (39.7 *vs.* 36.9; p<0.05). Similarly, no differences were observed in OPR (50% *vs.* 45.5%); pregnancy loss (20% *vs.* 23.4%) or negative β-hCG (30% *vs.* 31.2%) between donated or own oocytes, respectively (all p>0.05).

**Figure 1:**
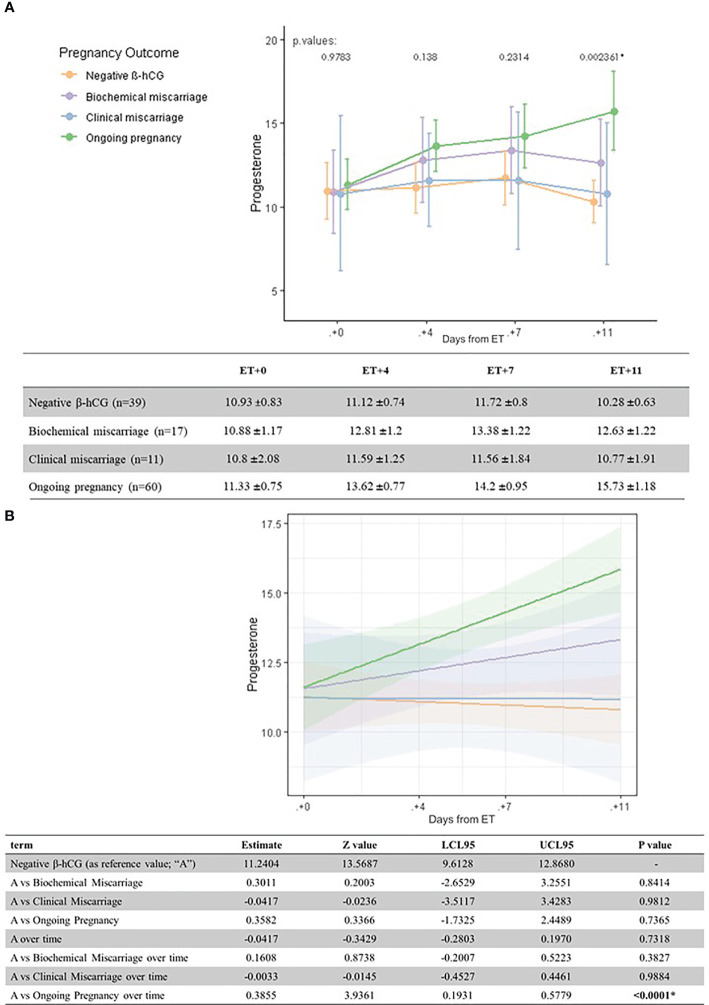
Univariate analysis **(A)** and linear regression model **(B)** of serum P levels (ng/ml) according to pregnancy outcome and the day after ET.

**Figure 2:**
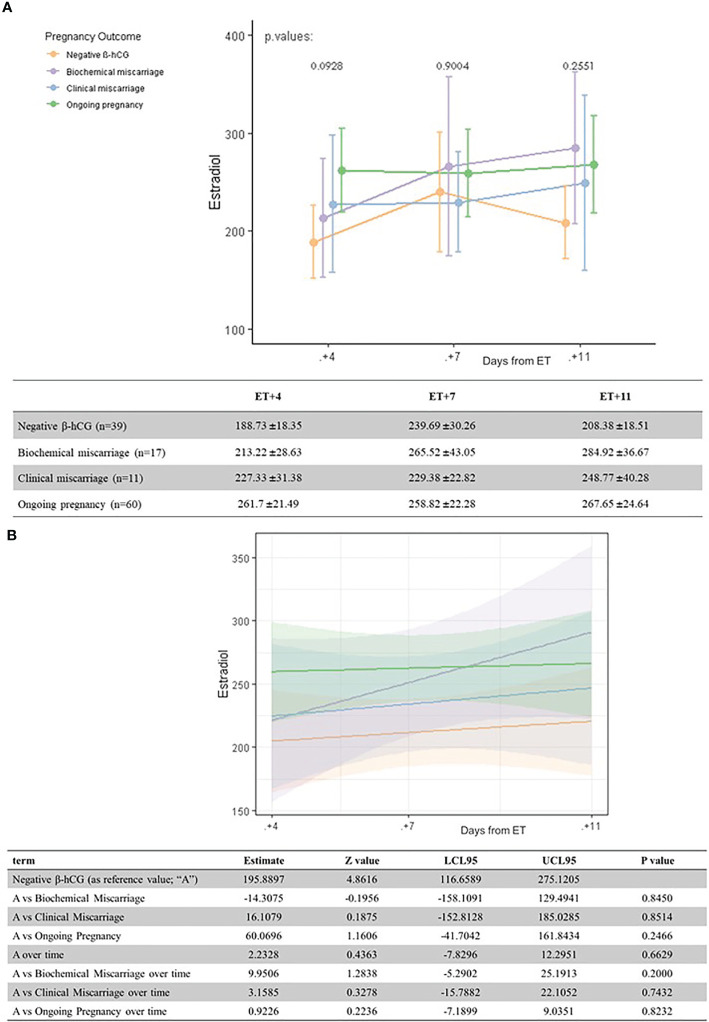
Univariate analysis **(A)** and linear regression model **(B)** of serum estradiol levels (pg/ml) according to pregnancy outcome and the day after ET.

The mean levels of serum progesterone and estradiol throughout the mid and late luteal phase are shown in **Figures 1** and **2**, respectively. Results are shown according to the final pregnancy outcome (defined as negative β-hCG, biochemical miscarriage, clinical miscarriage or ongoing pregnancy). The behavior of serum P according to the pregnancy outcome followed the same pattern in both own or donated oocyte cycles (**Supplementary Figure 1**). **Table 1** shows mean serum P levels throughout the late luteal phase regarding pregnancy outcome within each one of the three scenarios for oocytes’ origin: oocyte donation, own oocytes without PGT-A, and own oocytes with PGT-A. Mean ages of each one of these subgroups are also displayed in this table.

**Table 1:** Mean serum P levels throughout the late luteal phase and outcome regarding oocytes’ origin: oocyte donation, own oocytes without PGT-A, and own oocytes with PGT-A.

	Age of oocytes	Age of recipients	Outcome	Serum P ET+4	Serum P ET+7	Serum P ET+11
Oocyte donation (n = 56)	24.5 ± 1.9	39.3 ± 4.0	Ongoing pregnancy (n = 27)	12.0 ± 4.1	13.2 ± 4.9	15.8 ± 9.7
			Negative ßhCG (n = 18)	11.9 ± 5.6	12.5 ± 10.8	10.8 ± 4.2
			Biochemical miscarriage (n = 8)	12.5 ± 4.8	13.7 ± 4.8	13.8 ± 5.6
			Clinical Miscarriage (n = 3)	10.9 ± 3.7	8.3 ± 4.3	5.9 ± 2.9
Own oocytes without PGT-A (n = 30)	34.1 ± 3.7		Ongoing pregnancy (n = 14)	16.3 ± 7.5	17.2 ± 9.8	16.8 ± 9.8
			Negative ßhCG (n = 7)	9.8 ± 3.0	10.5 ± 3.3	9.5 ± 4.3
			Biochemical miscarriage (n = 5)	13.9 ± 5.1	13.1 ± 5.3	12.0 ± 4.6
			Clinical Miscarriage (n = 4)	10.0 ± 4.1	13.1 ± 8.9	14.6 ± 8.2
Own oocytes with PGT-A (n = 41)	38.3 ± 3.15		Ongoing pregnancy (n = 19)	13.9 ± 6.5	13.5 ± 8.1	14.8 ± 8.3
			Negative ßhCG (n = 14)	10.8 ± 3.9	11.3 ± 4.6	9.9 ± 3.6
			Biochemical miscarriage (n = 4)	12.2 ± 6.3	13.2 ± 6.6	11.1 ± 5.0
			Clinical Miscarriage (n = 4)	13.9 ± 4.0	12.5 ± 4.4	10.6 ± 4.2

Mean ages of the oocytes (and ages of the recipients in those cases of oocyte donation) are shown in the second column.

Serum P levels increased as luteal phase advanced in patients with an ongoing pregnancy. The one-way ANOVA test showed statistically significant differences on serum P levels depending on pregnancy outcome on day ET+11, in particular between ongoing pregnancies and negative β-hCG cases (*p* = 0.002).

When comparing mean serum P levels in ongoing pregnancies *versus* negative β-hCG cases, there was a significantly higher level on ET+4 (13.6 ± 6.0 ng/ml *vs.* 11.1 ± 4.6 ng/ml, p=0.03) and ET + 11 (15.7 ± 1.2 *vs.* 10.3 ± 0.6 ng/ml, respectively; *p* = 0.000). On ET+7, serum P levels were also higher in ongoing pregnancies *vs.* negative β-hCG cases (14.2 ± 0.9 *vs.* 11.7 ± 0.8 ng/ml, but did not reach statistical significance (p=0.07) (**Figure 1**).

Serum estradiol levels were not related with the pregnancy outcome at any moment of the luteal phase (all differences *p >*0.05) (**Figure 2**).

**Figure 3:**
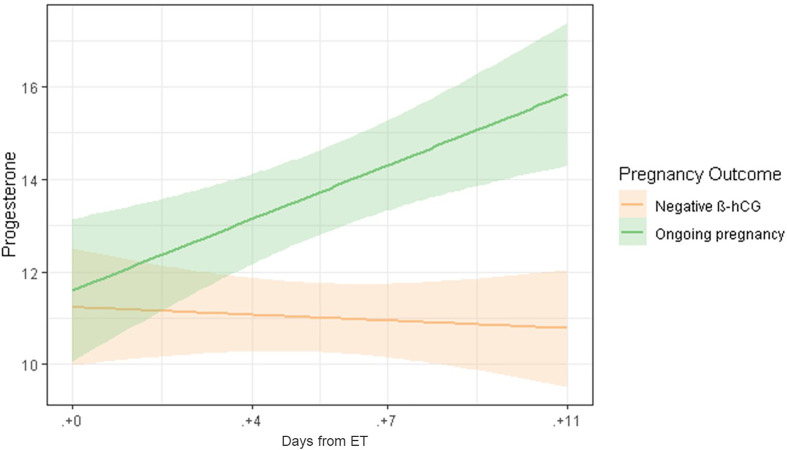
Linear regression model of serum P levels throughout the late luteal phase, comparing ongoing pregnancies (green) and negative β-hCG cases (orange).

A linear regression model confirmed the significant difference in serum P levels as we move through the late luteal phase between ongoing pregnancies and negative β-hCG cases (**Figures 1** and **3**).

**Figure 4:**
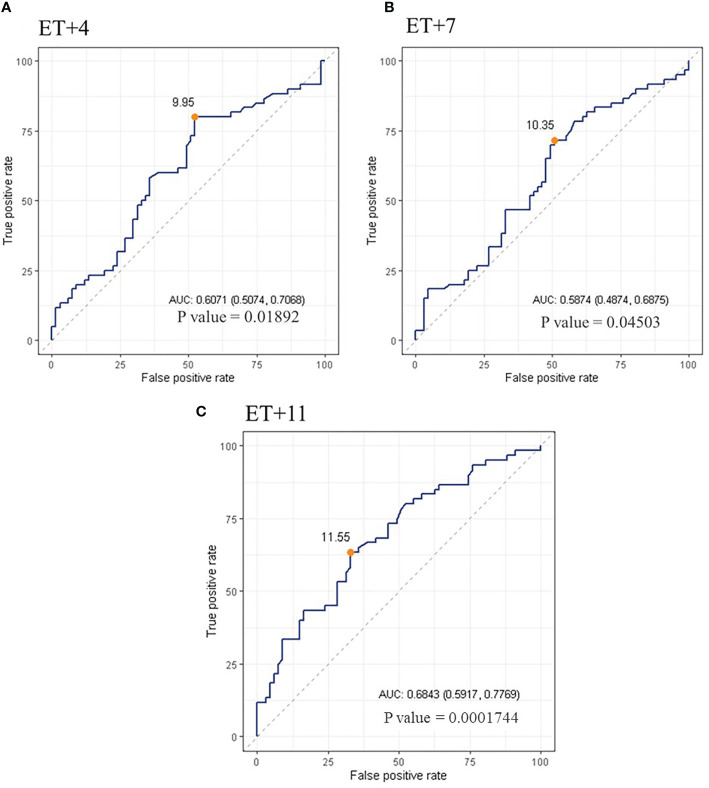
ROC curve of serum P levels on day 4 **(A)**, 7 **(B)** and 11 **(C)** after ET. The orange dot points out the cut-off value calculated yielding the best sensitivity and specificity.

The ROC analysis indicated that serum P levels were predictive of ongoing pregnancy, being the AUC (95% CI) = 0.61 [(0.51-0.71); p=0.01892] on ET+4; 0.59 [(0.49-0.69); p=0.04503] on ET+7; and 0.68 [(0.59-0.78); p=0.0001744] on ET+11. The curve defined an optimal cut-off value for serum P of 9.95 ng/ml on day ET+4, with a sensitivity of 0.80 and a specificity of 0.48; a value of 10.35ng/ml on day ET+7, with a sensitivity of 0.72 and a specificity of 0.49; and a value of 11.55ng/ml on day ET+11, with a sensitivity of 0.63 and a specificity of 0.67 (**Figure 4**).

Pearson’s coefficient (r) showed a significant association (*p*<0.05) of serum β-hCG levels with serum P on ET+4 (r = 0.20), serum P on ET+7 (r = 0.19) and serum P on ET+11 (r = 0.25).

## Discussion

According to the results of the present study, the association between serum P levels and pregnancy outcome is observed during the whole mid-luteal phase. Those patients with an ongoing pregnancy showed higher levels of serum P than the rest of patients, and there was an increasing trend throughout luteal phase days. This behavior was not observed in the other groups (patients with a negative result or pregnancy loss).

Ongoing pregnancies showed the highest serum P levels on each determination day of the late luteal phase. Nonetheless, this association was more evident when comparing ongoing pregnancies and negative β-hCG cases on day ET+11 (**Figure 1**). This is in accordance with a previous study in artificial cycles (3), which suggested that serum P could have a predictive value on pregnancy outcome when measured on the day of the pregnancy test. The correlation between serum P levels on the day of the pregnancy test and pregnancy outcome has been also studied in stimulated cycles, with contradictive results. While some authors suggest that serum P can have a predictive role (8, 9), others could not find this association (10). The difficulty in these stimulated cycles relies on the fact that the endocrine profile depends on the ovarian response, type of trigger and luteal phase support (11–14), and this could influence the interpretation of results. On the contrary, the advantage in the artificial cycle is that the source of progesterone during the luteal phase comes exclusively from the exogenous administration, as we always check the absence of spontaneous ovulation before introducing progesterone. This means that serum P in artificial cycles is reflecting the absorption capability of the patient to this hormone.

Interestingly, a linear regression model confirmed the significant time effect on serum P levels according to pregnancy outcome, in particular when comparing ongoing pregnancies and negative β-hCG cases (**Figure 3**). It is worth noting the odd behavior of serum P levels in biochemical and clinical miscarriages. The later showed lower serum P levels, very close to negative β-hCG cases, even though gestational loss does not occur until later in pregnancy. Notably, we observed wide SD ranges of P in patients with miscarriage that can be attributed to the low number of cases. This difference, although not significant, deserves further study. Patients who ended up with a biochemical miscarriage showed a trending increase of serum P levels until ET+7, but decreased on ET+11, in comparison with ongoing pregnancies, which continued increasing.

This later finding, deserves further comments. In natural and stimulated cycles, serum P levels may indicate the response of the corpus luteum to the hCG secreted from the placenta in the early pregnancy period (15). This production of P remains present until approximately 7-8 weeks of gestation, when the luteoplacental shift occurs and the placenta takes over endogenous steroid synthesis (16). On the contrary, in artificial cycles, serum P levels should remain quite constant during the luteal phase, depending exclusively on the absorption of exogenous P, due to the lack of a corpus luteum.

Some pharmacokinetic studies have demonstrated that mean serum P levels reach a plateau after 12 hours, with almost steady state concentrations thereafter (17). If progesterone intake is maintained, these levels should not be altered unless other factors influence the absorption potential. For example, in postmenopausal women, estrogen priming results in increased vaginal progesterone absorption (17), as estrogens can overcome the problems related to thinner and atrophic vaginal mucosa in older women. In our study, all patients were under estrogen treatment, and we could not observe any relation of serum estradiol levels with pregnancy outcome or serum P levels.

Having said this, the fact that serum P levels tended to increase in patients with an ongoing pregnancy as luteal phase advanced could be expected in ovulatory cycles, but not in artificial cycles. The remaining question is if these higher results are the cause or the consequence of the final optimal pregnancy outcome.

Some scientifical evidence could support that higher levels could be the “cause” of the final result. It has been recently demonstrated that low serum P levels on the mid luteal phase are related with lower ongoing pregnancy and live birth rates (6). Thus, those patients with higher basal serum P levels are more prone to have better results. We speculate that, at the same time, the pregnancy itself might induce modifications on the vaginal mucosa or increase the vascularization at this level, enhancing the vaginal absorption of progesterone. This hypothesis could be valid only when vaginal P is used, based on our results. In fact, when using oral dydrogesterone in artificial cycles, progesterone starts to increase in pregnant women marginally on ET+16 and evidently from ET+23 onwards, meaning that the increase in serum P levels occur later (18). Thus, when using synthetic progesterone, the changes observed in this study are not shown.

A preliminary study conducted in oocyte donation recipients under artificial cycles for endometrial preparation suggested that the onset of placental steroidogenesis surrounded the 5th gestational week (19), while a more recent study found that endogenous placental activity started on 5^th^-6^th^ pregnancy week, differing significantly according to the pregnancy outcome (7).

However, different serum P levels are found in different patients after the exact same dose of exogenous P for luteal phase support, and these different P levels are related to different pregnancy outcomes. On the one hand, the inter-variability in P absorption may lead to impaired embryo implantation and subsequent pregnancy maintenance in those patients no capable of absorbing sufficient P. On the other hand, these authors speculate that an impaired or absent embryo implantation may hamper P absorption, or it may even interfere with a yet unknown mechanism of early P production triggered by the placenta, which may help with the sustention of serum P levels in ongoing pregnancies, but this is yet to be demonstrated.

Regardless of the origin, serum P levels throughout the late luteal phase may help us to distinguish between an ongoing pregnancy or an implantation failure. However, the minimum threshold point established for the day of ET (8.8 ng/ml) cannot be extrapolated to the rest of the luteal phase, as the current study shows serum P levels above this value on ongoing pregnancies after ET. The ROC analysis performed suggests optimal cut-off values for serum P on each day of the late luteal phase; all of them being higher that the critical threshold described previously. Further larger studies should be performed in order to establish a serum P threshold point in the late luteal phase, capable of discriminating between successful and unsuccessful implantation. However, it remains unknown if the unsuccessful implantation situation could be solved somehow once it has been detected on day ET+4.

Regarding estradiol levels, their relation with pregnancy outcome has been previously described in stimulated cycles (20). In our study, interestingly, serum estradiol levels increase more rapidly in biochemical miscarriages as they progress throughout the luteal phase, leading to the highest values on days 7 and 11 after ET. However, the wide CI suggests this may be due to a large variability in estradiol values, rather than to a specific causal effect.

It is plausible that both high P and β-hCG levels on day ET+11 might yield the best outcome, as it has been already demonstrated in stimulated cycles (8, 10). Further clinical studies focusing on this specific aim with a larger sample size are needed to demonstrate this.

In conclusion, the results of this preliminary study suggest that serum P might be playing an important role not only during implantation, but also in pregnancy maintenance. Higher P values during the late luteal phase are related to better pregnancy outcomes, even though the more reliable determination happens to be on day ET+11. The limitation of this study is that the number of miscarriages is not high to draw strong conclusions, but is a starting point to investigate on this. It is still unknown if serum P levels just reflect or are the cause of the related pregnancy outcome. In any case, further studies should be performed in order to elucidate if increasing the dose of exogenous P would benefit those patients with lower serum P levels in the late luteal phase, as well as the specific role of the combined action of P and β-hCG on pregnancy maintenance.

## Data Availability Statement

The raw data supporting the conclusions of this article will be made available by the authors, without undue reservation.

## Ethics Statement

The studies involving human participants were reviewed and approved by Comité Ético de Investigación Clínica del Instituto Valenciano de Infertilidad-IVI. The patients/participants provided their written informed consent to participate in this study.

## Author Contributions

EL and EB significantly contributed to the study conception and design, performed statistical analyses and data interpretation, and drafted the article. CR-V contributed to data interpretation and drafted the article. GM contributed to patient´s inclusion and data collection. All authors contributed to the article and approved the submitted version.

## Funding

CR-V received a grant from the Spanish Ministry of Science, Innovation and Universities in 2019 for the National Programme for Training University Lecturers (FPU). All authors declare no conflict of interest.

## Conflict of Interest

EB has received honoraria from Ferring B.V., Gedeon Richter, Merck and Roche; acted as a paid consultant for Ferring B.V., Merck, Gedeon Richter, Roche and Abbott; and reports research cooperation with Gedeon Richter. EL serves on the speaker bureau for Ferring B.V., Merck, MSD, IBSA and Gedeon Richter.

The remaining authors declare that the research was conducted in the absence of any commercial or financial relationships that could be construed as a potential conflict of interest.

## Supplementary Material

Supplementary Figure 1 Linear regression model of serum P levels throughout the late luteal phase, comparing ongoing pregnancies (green) and negative β-hCG cases (orange) differentiating cycles with own or donated oocytes.
