# Supplementary figures and images for: Serum Progesterone Profile Across the Mid and Late Luteal Phase in Artificial Cycles Is Associated With Pregnancy Outcome
Source: Front Endocrinol (Lausanne). 2021 Jun 10;12:665717. doi: 10.3389/fendo.2021.665717 (PMC8224169; doi:10.3389/fendo.2021.665717)

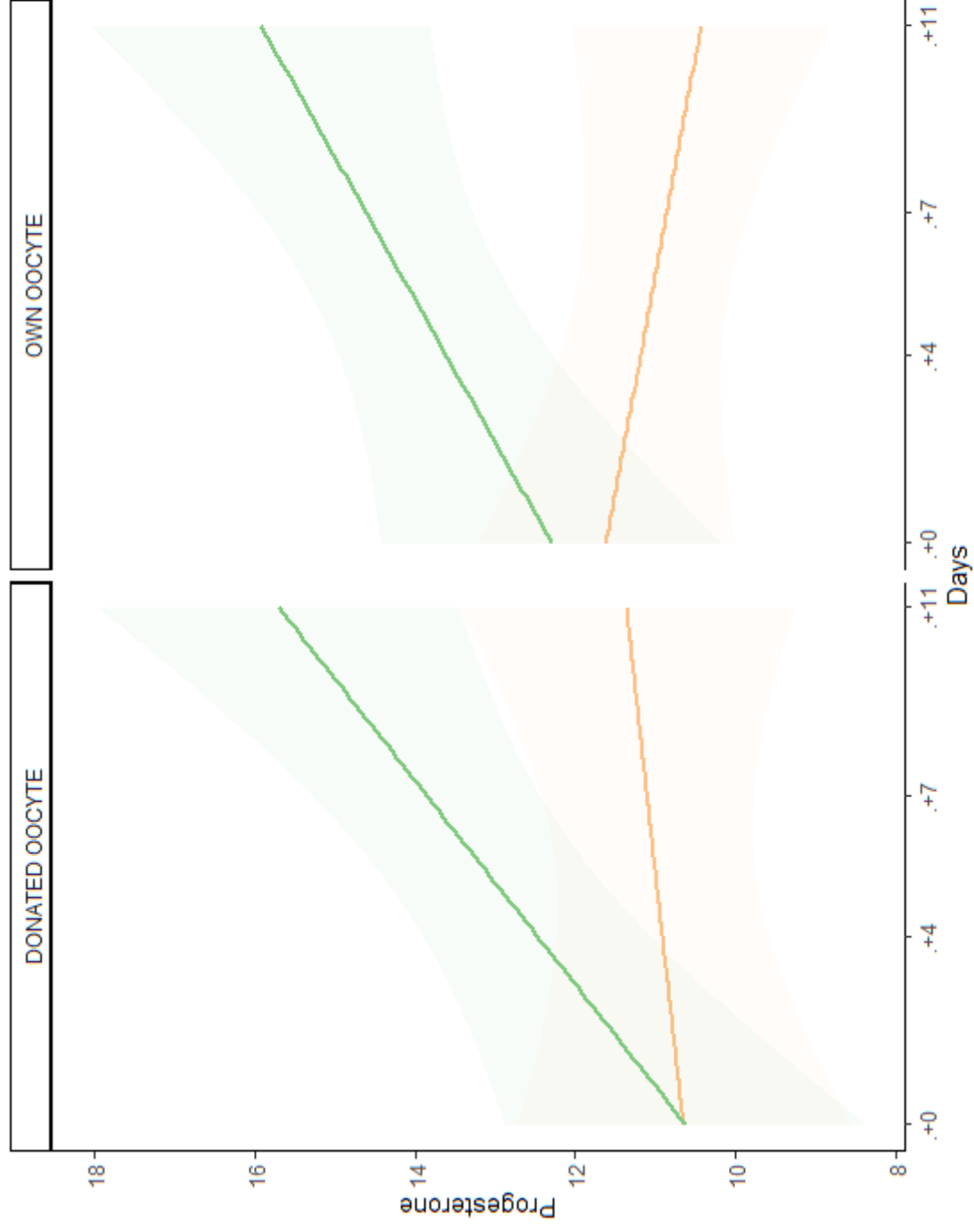

Supplement: Supplementary Figure 1 — Linear regression model of serum P levels throughout the late luteal phase, comparing ongoing pregnancies (green) and negative β-hCG cases (orange) differentiating cycles with own or donated oocytes. [file DataSheet_1.pdf]
